# A Smart City Application: A Fully Controlled Street Lighting Isle Based on Raspberry-Pi Card, a ZigBee Sensor Network and WiMAX

**DOI:** 10.3390/s141224408

**Published:** 2014-12-18

**Authors:** Fabio Leccese, Marco Cagnetti, Daniele Trinca

**Affiliations:** Dipartimento di Scienze, Università degli Studi “Roma Tre”, Via della Vasca Navale 84, Rome 00146, Italy; E-Mails: ing.marco.cagnetti@gmail.com (M.C.); cico80@gmail.com (D.T.)

**Keywords:** control systems for smart grids, lighting system, wireless networks for smart grids, ZigBee, sensors, Raspberry-Pi, ARM processor

## Abstract

A smart city application has been realized and tested. It is a fully remote controlled isle of lamp posts based on new technologies. It has been designed and organized in different hierarchical layers, which perform local activities to physically control the lamp posts and transmit information with another for remote control. Locally, each lamp post uses an electronic card for management and a ZigBee tlc network transmits data to a central control unit, which manages the whole isle. The central unit is realized with a Raspberry-Pi control card due to its good computing performance at very low price. Finally, a WiMAX connection was tested and used to remotely control the smart grid, thus overcoming the distance limitations of commercial Wi-Fi networks. The isle has been realized and tested for some months in the field.

## 1. Introduction

The Smart City (SC) paradigm helps renovate the traditional city concept. In fact, it is possible to realize and develop efficient demand-side strategies integrating the monitoring and automation features ensured by intelligent devices and their communication apparatuses typically used in many applications. Within this concept, public lighting, being a great electrical energy consumer, has recently been attracting the interest of the research community. Scientists, combining the SC paradigm with alternative energies and new lighting technologies, are conceiving systems previously unimaginable, which can increase the efficiency obtaining considerable energy consumption savings and consequently money savings [1–4].

This article shows how an isle of lamp posts, used to light a crossroad and placed in an area not reached by ADSL lines and 3G signals, can be much more efficiently managed by applying the smart city principles. The realized application changes the way of conceiving the isle: sensing the transit of a car approaching the crossroad and controlling it by an efficient management strategy, a typical power grid, like the isle of the lamp posts, is transformed in a smart city application.

The goal is obtained combining an efficient management strategy with new technologies. The strategy divides the complex structure of the isle into layers which are linked by communication layers. Each layer uses innovative technologies to make possible the application of the smart city principles. For example the lamp post exploits some innovative technologies both for the supply (photovoltaic panel) as well as for the control (ZigBee network and RaspBerry-Pi). Moreover, to provide a robust system also able to face and overcome the absence of Asymmetric Digital Subscriber Line (ADSL) or 3rd Generation (3G) communication, that in some parts of the city might not available, we used WiMAX technologies that allowed us to fully control the isle from a remote station.

The choice to test our system in this application is strongly joined to the road safety problem. In fact, the lighting of crossroads is fundamental to ensure the good night vision of typically hazardous points of the road where fatal crashes often happen. Based on this application, an economical analysis has also been realized to show how after the breakeven point is achieved, the system is more convenient with respect to older technology.

## 2. State of the Art

In this area, the efforts are focused on the use of alternative energies for the power supply of new lighting technologies [5–7], which allow obtaining considerable energy savings. Within lighting technologies, Light Emitting Diodes (LEDs) assure the possibility of switching on the lamp without the preheating typical of halogen ones; a very high lighting efficiency; low power consumption; a superior life time and quick switching times not comparable to those of older technologies (only incandescent lights have a lower lighting time, but with very big power consumption and the shortest time life); less sensitivity to transient phenomena, which have a big impact on other technologies, allowing thousands of lightings without the risk of lamp failure. These innovative characteristics allow development of a new remote-control system based on intelligent lamp posts that also send information to a central control system in order to simplify management and maintenance issues [1–4] also using holistic and bottom-up design strategies [1]. Within these researches, a relevant topic is the use of new sensor networks (wireless and not) and communication technologies, both to locally manage the lamp posts, but also sensor networks, and to send data towards a remote center [1–5,8–17].

Based on the best technologies and on the SG paradigm, our proposal describes a solution for an extremely diffused hostile scenarios, often neglected by the municipalities, but which have an enormous social relevance, and where both the mains and the internet signal provided by the most diffused communication technologies (ADSL and 3G) are not available.

## 3. The Layers Architecture

The architecture of the system has been conceived as a hierarchical layers structure for the full control of “unities” made up of sensors, actuators and local control cards. Each layer performs some control activities and transmits information to the other layers. The architecture foresees three layers called apparata, local control and remote control.

**Figure 1.**
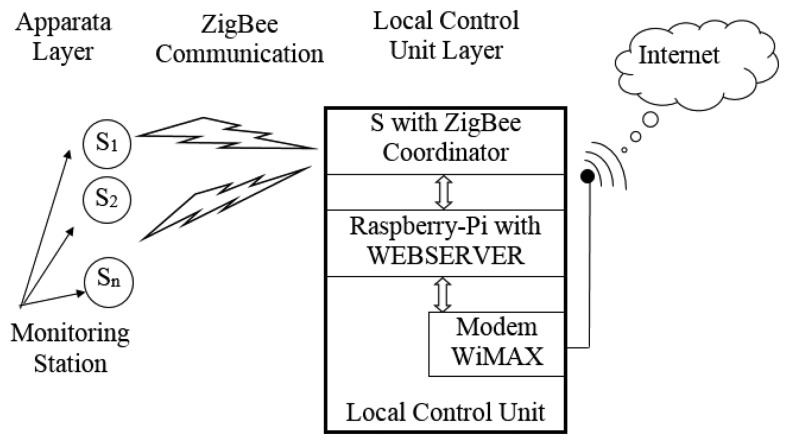
Block scheme of the system architecture.

In the “apparata layer” we consider all the hardware and software designed for the autonomous and automatic control of the single apparatus or unit. This layer communicates with the higher one and *vice versa* using a ZigBee mesh wireless tlc network particularly suitable for closed devices and low bit rates [3]. The local control unit collects the data received from the single units to check their activity and functionality. It is realized with a RaspBerry-Pi control card, which collects and locally elaborates data from the single apparatus verifying that there are no problems. If a problem(s) occurs, the card sends an error message on the internet exploiting the upper layer. The latter is realized with a WiMAX trans-receiver, which is connected, *via* radio link, to the World Wide Web. Figure 1 shows the architecture of the system.

## 4. Technologies and Devices

Before analyzing the realized application, the three technologies and devices used in this work are briefly described.

### 4.1. ZigBee

ZigBee is a standard for wireless communication based on the IEEE 802.15.4 protocol. It is used to connect several devices together creating a mesh network with no central powerful device, but with distributed nodes able to transfer data throughout intermediate motes. Table 1 shows a comparison between the most famous technologies used as wireless communication. As the table shows, both Wi-Fi and the Bluetooth have higher bit rates than ZigBee, but the higher power consumption and the lower number of devices making up the network suggest that the ZigBee technology is the best candidate to create sensor networks for applications where the power consumption must be as low as possible. Furthermore, studies proved that the ZigBee is more affordable in terms of power consumption and costs than competitive technologies [3,18–20].

The communication between the lamp posts in the isle has been achieved using the ZigBee standard. This uses a mesh topology with a coordinator, routers (also called primary) and end device nodes (also called secondary); the first controls the formation and security of networks, the second extend the range of networks while the last perform specific sensing or control functions. Each lamp post has a Tx/Rx to get or send information and control commands. Furthermore, the lamp posts closer to the roads, used to approach the intersection, are also connected to a presence sensor card. The secondary nodes will send their status to the coordinator lamp post throughout the mesh network and receive back commands. Thus, the power consumption is limited to short time frames, since there is no continuous communication between nodes.

The networked communication is realized using the XBee radio modules by MaxStream which are already equipped with on-chip antenna [21–23]. These modules have been chosen because they have a good operational range for the application reaching tens of meters indoors and hundreds of meters outdoors. Thanks to its high radio sensitivity the XBee module has a low probability to receive corrupted packets (less than 1%). Furthermore the reported tests demonstrated that these performances are achieved without sacrificing power consumption. In fact, the current necessary to operate the two-way radio transmissio, is approximately 50 mA with a 3 V DC source. To ensure further power reduction, the sleep mode ensures a current draw of less than 10 μA.

### 4.2. RaspBerry-Pi

The role of the Coordinator Lamp Post is to integrate the computing part of the system. For this aim, the Raspberry-Pi card (or Pi following) has been adopted which ensures high computing power and interconnectivity with other devices such as the WiMAX modem [24–27].

Raspberry-Pi is a low-cost (≈25 €) basic computer contained on a credit-card size circuit board and featuring ports for HDMI, USB 2.0, composite video, analog audio, power, Internet and SD card. The computer runs entirely on open-source software and is able to run several applications such as: spreadsheets, word-processing, games, and high-definition video playback.

Other devices, as ARDUINO [28] or Beaglebone [29] could be used; Table 2 shows some principal characteristics of these devices. As usual, choosing which board you want depends on the type of intended project, and one's programming experience. If one wants to create a hardware project then the Arduino is by far the best choice. The analog inputs and PWM outputs add a whole spectrum of compatibility the Pi cannot provide natively. In addition, the large about of I/O pins let one connect multiple sensors and feedback components. The Arduino however is not as powerful as the Pi, so it has no proper audio, video or internet out-of-the-box (you can however add basic functionality for this). The Arduino can send data to a PC or Pi, over serial connections, and one can then create a program to read this data and do something useful.

If one wants to create a software project then the Pi and the Beaglebone are the way to go. The audio, video and internet capabilities make them the winners in this aspect. There is no need to attach external components, so there is no real need to learn electronics and they have the possibility to easily drive both 3G modems and WiMAX ones. Among them, the lower power consumption and lowest cost favor the RaspBerry-Pi, making it the best candidate for SC applications.

The Raspberry is the hub of the system since it allows the visualization of the status of the entire lighting system. It is connected to the local monitoring station *via* the ZigBee network to manage the different lamp posts. Furthermore, it is able to receive information from the secondary lamp posts and from the presence sensor cards allowing monitoring the isle. Together, the Raspberry and the local monitoring station realize the Coordinator Lamp Post base control station, which receives information about the status of the lamp posts.

### 4.3. WiMAX

Worldwide Interoperability for Microwave Access (WiMAX) is the radio communication technology used to connect the Local Control Unit with the World Wide Web. It is based on the IEEE 802.16 standard, with the intent of deliver Internet connectivity to areas where a normal DSL service is still difficult and expensive to install or in parts of cities not reached by 3G signals [5,30].

The reason for choosing WiMAX is that in some parts of the city, e.g., very far from the downtown, sometimes, there is very scarce penetration of telecommunication lines or of the radio communication coverage. In the best cases with clean line of sight (LOS) the radio link can cover up to 70 km [30]. Other technologies could be used to realize the remote communication such as 3G, satellite communication, WiFi or ADSL, but, as Table 3 shows, none offer the specific abilities of WiMAX. Excluding ADSL that needs a cable whose placement could be extremely expensive in these areas, and WiFi whose coverage is extremely short (less than 100 m), satellite communications offer worldwide coverage, but its subscription is usually much more expensive than a WiMAX or a 3G. The latter, always because is not profitable for the provider to create a 3G network, is not always active in these areas. For these reasons, WiMAX is a good candidate for this application. This would also allow diffusion of the SC paradigm in areas not properly considered as “city”.

In our system, we installed a base station (BS), spreading WiMAX single carrier TDD service at 3.4845 GHz center frequency and a bandwidth of 10 MHz. The BS transmitting antenna is an Argus tilt panel antenna model SPPX310M (65 deg Horizontal cut, 6.5 deg Vertical cut) [31].

The total transmission power is 55 dBm EIRP given by the output signal of 37 dBm and an antenna gain of 18 dBi. The antenna was placed on the roof of a building at 30 m from ground level. To communicate between the main lamp post and the BS, we used a Huawei branded HES-319M outdoor WiMAX Customer Premise Equipment (CPE) [32], equipped with a 3.5 GHz 45° Cross-Polarization built-in directional antenna, with a maximum power at antenna port of 26 dBm and 14 dBi antenna gain. This allowed us to send reliably data at high speed. Furthermore, it avoids any type of interference with the ZigBee standard due to its different operating frequency.

The WiMAX module uses 64QAM5/6 modulation for the downlink when the CINR is higher than 30 dB and 16QAM3/4 for the uplink [33,34]. Instead, when the CINR is lower than 10 dB, the link was still possible, but using a QPSK1/2 modulation because it is more robust at higher noise levels.

The CPE is Power over Ethernet (PoE) 802.3 a/f compliant and it runs at 48 VDC, so a DC-DC step-up boost has been required to make the CPE working in our system. We used a LTC3863 mounted on its demonstration circuit 1286 A in order to boost up the 12 V from solar panel to the 48 VDC needed at the CPE PoE connector.

## 5. The Application

**Figure 2.**
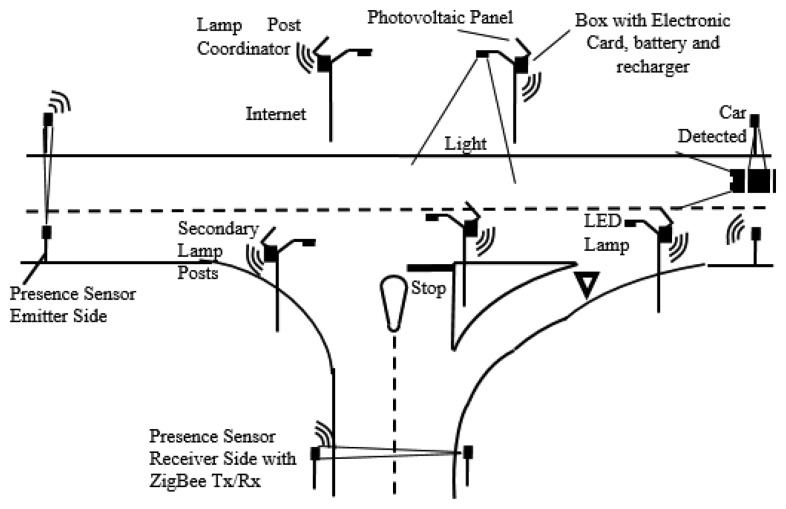
Schematic image of the on street system.

To widely test the idea, an isle of lamp posts, as shown in Figure 2, has been designed and realized.

**Figure 3.**
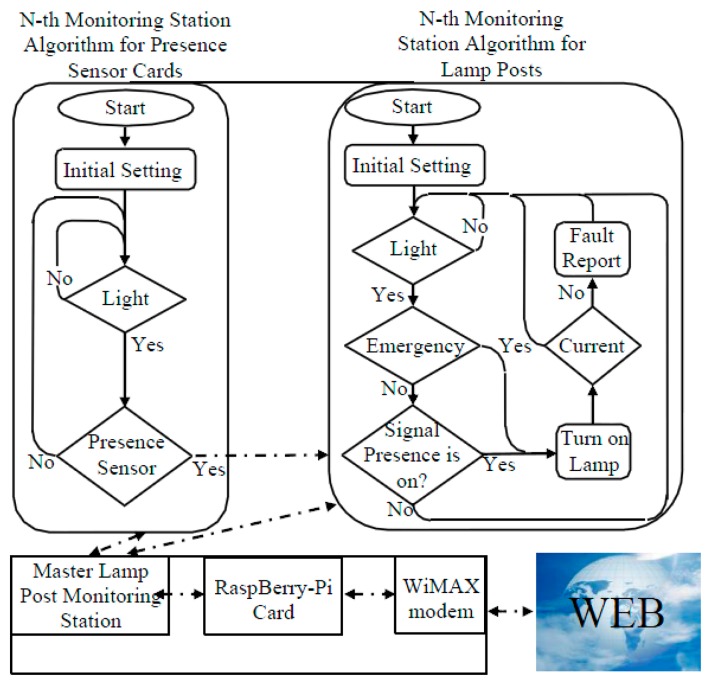
Management strategy of the Smart Grid.

Through a flowchart, Figure 3 shows the management strategy of the isle.

The electronic card checks if the sunlight is lower than a fixed limit [3]; in this case, it waits until the sensors signal the presence of a car or a pedestrian. When this happens, the control unit switches on the light for a fixed time, the current sensor starts the measurements and, in case of a fault detection, an alarm is sent to the master lamp. If no fault is detected, the microcontroller stores the current values. During the night the Coordinator Lamp Post requests data from the secondary lamp posts and makes them available on the Internet. In the case of people in difficulty, which need light for their activities, they will be able to switch on the lights by pressing one of the emergency buttons located in specific locations at the crossroad. The electronic card, also called monitoring station, is used in two versions: the first detects the presence of a vehicle and is called Presence Sensor Electronic Card, while the second manages the lamp post and is called Monitoring Station for Lamp Posts.

The first are placed near the road at about 45 m from the crossroad. They are enabled when the sunlight is lower than a fixed limit. They read a photoelectric sensor and if its light beam is interrupted by the passage of a car, the control card sends a wireless signal to the isle of lamp posts by the ZigBee network. When this signal is received by the Monitoring Stations for Lamp Posts and it are equipped with a ZigBee Tx/Rx, they switch on the light.

The monitoring station is realized with the use of a PIC 16 f 688 microcontroller that also manages the wireless transmissions. The lamp post management is thus realized, but for intelligent management, the maintenance needs require a wider and deeper control of the lamp posts. To meet these requests, a current sensor, directly mounted on the lamp posts and read by the Monitoring Station, provides information about lamp post activity and power consumption. This information is stored in the PIC memory and, if requested by the next layer, is sent to it.

The second layer communicates with the first layer by the ZigBee network and collects data coming from each lamp post. The collected data are then available for the analysis of power consumption and for monitoring the lamp activity. Obviously, if the n-lamp post does not communicate or sends wrong data, this layer will send an intervention request toward a remote operator to solve the problem.

**Figure 4.**
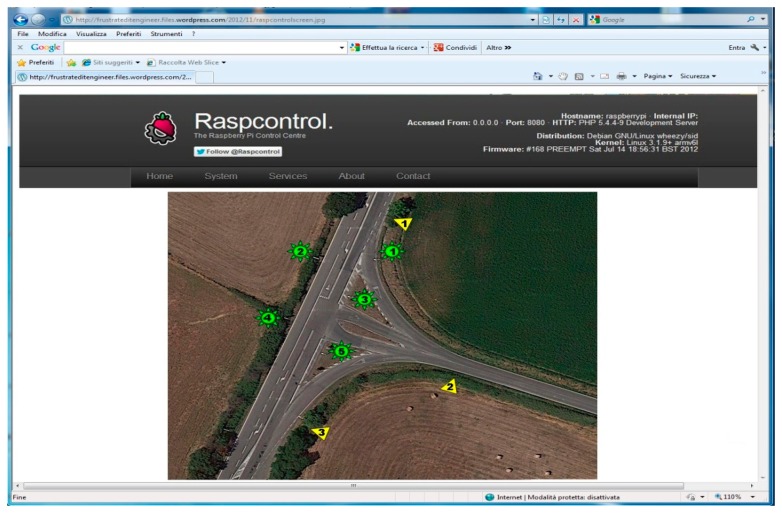
Lamp control system GUI and measurement of power consumption.

The second layer uses a RaspBerry-Pi card [24–27]. This is placed only in a lamp post called Coordinator, (another lamp post equipped with the RaspBerry-Pi may cause redundancy) and it is connected to the lamp post Monitoring Station. The latter makes possible the communication between the RaspBerry-Pi and the other lamp posts realizing the communication between the second and the first layer. The RaspBerry-Pi plays a role of webserver collecting data from each lamp post and displaying them. The third layer uses the RaspBerry-Pi to send these data to the World Wide Web. A graphical interface (Figure 4) shows the information on the lamp posts' activities.

**Figure 5.**
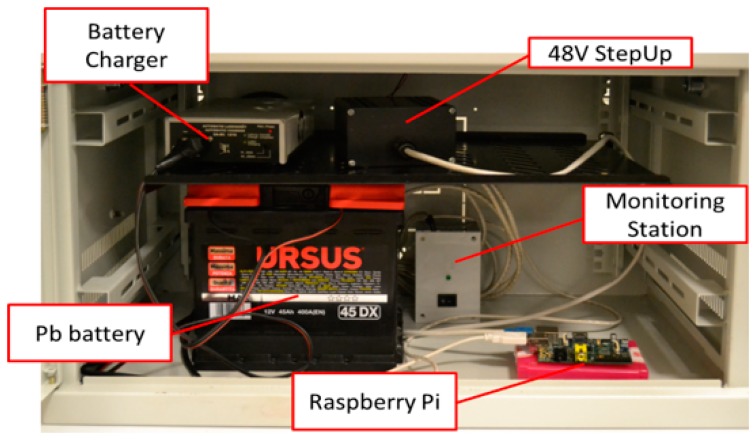
The system placed in a shelter for laboratory tests.

All this makes possible an easy control of the lamp posts from a remote station and can allow an easy scheduling of any maintenance actions by the service engineer. Figure 5 shows the system placed in a shelter for laboratory tests.

**Figure 6.**
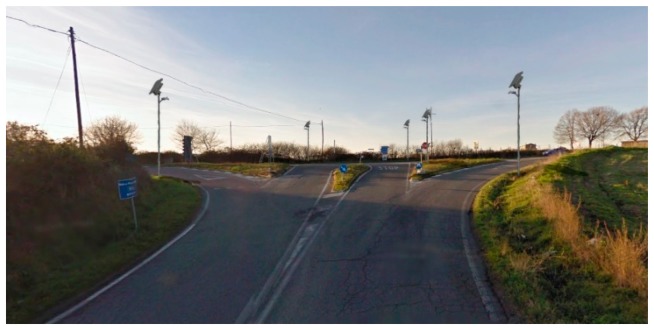
Test system in the field.

Figure 6 shows the operational test system working in real conditions.

## 6. The Lamp Post

The lamp posts use, as new technologies, Light Emitting Diodes (LEDs) for the illuminating lamps and photovoltaic energy (PV) to supply the power. The use of these technologies is known in the literature, but a preventive traffic study allowed precisely defining the load allowing the correct dimensioning of the PV elements size.

The choice to supply the lamp posts of an alternative energy source is imposed by the absence of mains in the area where the isle is placed, and strongly suggests the use of this kind of energies in areas where the mains is far away. In fact, it would be very expensive (requiring copper wires and civil engineering works) to connect the area. Among the alternative energies, the absence of a constant wind, suggested to us that the best alternative energy to use is the photovoltaic one. PV systems are composed of a PV panel, a battery and a battery recharger [35].

The choice of the PV elements' size has been studied to match the operative conditions of the lamp posts. To evaluate the load, a preventive check of the car transit along the street has been made for four months during winter and spring when the nighttime has a longer duration. Every night we registered an average passage of about one hundred cars, often passing in group of two-four cars each time. For each transit of cars, we fixed 30 s for the lighting of the lamps so, the worst case provides that the total time the lamps need to be lit every night is about 25 min. Considering also anticipated some emergency situations, our system provides energy for one hour and a half. We also considered natural and weather conditions; in fact, we chose a larger battery to compensate for low sunlight for several days. Considering the loads, we have three different combinations:

(1) for the presence sensor card (0.2 A current consumption) the battery has a capacity of 6 Ah and the PV panel has a maximum power of 9 W–12 V; this assures a functioning of 10 h for three consecutively nights;
(2) secondary lamp posts use a 19 W–12 V PV panel and a battery of 10 Ah capacity able to ensure two hours per night of use for three consecutively nights;
(3) the coordinator lamp post has higher a consumption than the secondary ones because it has also the Raspberry-Pi (current consumption 0.5 A), which must be always connected to the Internet during the night and the WiMAX modem/router, which is activated only for two times during the night 15 min every time. To limit the consumption, the lamp section is managed like the secondary lamp posts, so the PV panel has a peak of 95 W–12 V and the battery a capacity of 48 Ah which assures an activity of 2 hours/night and the full activity for the hub for three consecutive nights. The modem average power consumption is about 20 W.

Obviously, the consumption, and consequently the PV panels, the batteries' sizes and the costs, would be at least three times higher without the intelligent management system; moreover, the lower weight produces less stress on the mounting poles.

## 7. Tests and Results

The system has been realized and tested in the field for some months to verify the overall functionality under variable real-life conditions. The measurements allowed us to calculate energy and cost savings compared to other older technologies.

### 7.1. Range Tests

The article [3] has shown the reliability of the communication between two or more ZigBee modules under different environmental conditions is satisfactory. Nevertheless, following the procedure shown in [3], we performed some tests directly in the field where the lamp posts and the presence sensors are in line of sight (maximum distance about 45 m). Standard Xbee modules with patch antenna from Digi-MaxStream have been used because from datasheet [21] and from [3] they seemed to satisfy the range needs.

According to [3], we perfomed several tests, each one foreseeing 10,000 transmissions both during clear weather and during rain. Using an appropriate adapter to simulate the retransmission and using the X-CTU software provided by Digi-MaxStream, we checked the transmissions verifying that the test packetd, sent through the network by an Xbee module, arrived at the coordinator lamp and were correctly returned back. Using the minimum transmission power available, we obtained the results reported in Table 4, with an average reliability of 100%.

### 7.2. Local Transmission Tests

Other tests have been designed and realized to verify the overall communication ability between the coordinator, the secondary lampposts, and Internet. The first test verified how the system reacts when there is a fault in a lamp post or a presence sensor. Simulating the absence of one of them the hub software alerts of the malfunction on its graphical interface.

The second test verifies the case of break-downs of the coordinator lamp post. If the fault is either on the electrical section or in the local communication section, the hub software again shows the problem. Instead, if the fault is either on the RaspBerry-Pi card or on the WiMAX section, the impossibility to connect to the website means either breakage or theft. A secondary coordinator, activated on another lamp, could solve this problem.

### 7.3. WiMAX Test

The previously described WiMAX system was used to perform several reliability tests of the WiMAX technology. Due to the decay of the signal intensity with the increase in the working distance or with the presence of obstacles leading to multi-path fading, the IEEE 802.16.1 standard [36] does not provide any data on the distance achievable with WiMAX devices.

Because our goal is to also serve areas placed some kilometers away from the BS, we decided to test essentially Line of Sight (LOS) scenarios to verify our system by mounting the receiving antenna on top of an extendable tripod and placing the system on the roof of a building, whilst the BS was on the roof of a building itself. For the non-line of sight (NLOS) cases, three typical obstacle situations can be considered: when there is a hill between the BS and the client, when there are trees and when there is vegetation. As demonstrated by [37], vegetation and foliage in particular, cause unstable radio links and affect a lot the global throughput of the system obliging the CPE to work at lower modulations. However our aim is to verify the reliability of the radio link rather than the maximum bandwidth achievable.

The considered radio parameters are the received signal strength indicator (RSSI) and the carrier to interference plus noise ratio (CINR). The first is a measurement of the total received power of the frame preamble and is used by the subscriber station to determine the received signal level from the BS [38]. The latter represents a measure of the quality of the WiMAX signal. The higher the value of the CINR, the more throughput a link can maintain. The standard 802.16 supports the link adaptive modulation and the channel coding (AMC) and, in order to maintain a constant bit error rate (BER) of 1 part per million, it adapts to signal degradation by dropping to a lower modulation [39].

The AMC is controlled BS side and it applies the Modulation and Coding Schemes (MCS) which defines the most suitable modulation rate for current radio channel, and leads to the highest data rate possible. WiMAX mainly uses quadrature amplitude modulation (QAM) with its more powerful 64-QAM5/6 in downstream; it is optional in upstream [26]. Maximum modulation in upstream is usually 16-QAM3/4. In Table 5 the test shows no packet loss until 12.9 km.

### 7.4. Measurements

As explained, the control system manages the isle providing the switch-on times (in minutes) and the absorbed currents of the lamps (in Amperes). Every control card of the lamp posts collects these data sending them to the coordinator when it requests them. After that, the coordinator control card provides to post the data on the website. To avoid data collisions, the coordinator calls the lamp posts one by one exploiting a specific identification number (ID) associated to each of them. Data collected during some autumn and winter months of 2013/14, showed the lamps absorb about 1.5 A during the activity for a period between 22.5 and 24 h per month.

### 7.5. Comparison with a Classic Lamp Posts

The absence of mains near the isle prevented us to making a direct comparison with a classical technology lamp post as for *i.e.*, in [3], so we designed this comparison following two ways: in the first our lamp post (called L1) is directly compared with another (called L2), similar to the first, but without the presence sensor control, so it was always switched on during the night; in the second, we realize a simulation considering the L2 supplied by mains (this lamp post is called L3). The results are reported in Table 6.

We performed this test in February 2014 and both the lamp posts used a 18 W LED technology, 1550 lumen, 84 lm/W luminous flux, powered by a solar panel and a battery. For L3 we assumed the lamp post was supplied by mains through a 12 V AC/DC converter.

Considering fixed the cost of the kWh of power (in Italy ≈0.2 € [40]) and that the first and second lamp posts have no power consumption, to find the break-even we used the following formula [3]:

(1) $$PC_{Z}-PC_{Y}+\sum_{i=1}^{x}\left(kWh_{Zi}-kWh_{Yi}\right)\cdot C_{kWh_{i}}=0$$

where PC is the plant cost difference between different lamp posts and it is a fixed cost; kWh is the number of kilowatt per hour used by the specific lamp post day by day; C_kWhi_ is the cost of kWh; it can change during a long period. The term x is unknown and represents the activity days necessary to reach the breakeven between the two different choices of lamp posts. As imaginable the first solution is always more convenient than the second, while the first becomes more convenient than the third after 56 months.

For the coordinator lamppost we need to add the cost of the WiMAX apparatus (80 €), while the Internet subscription cost is not considered because it is included in a WiMAX country public service. Moreover, there is a higher cost of the PV panel and of the battery equal to 80 €. Distributing this last cost between the five lampposts of the isle the breakeven is reached after 83 months. Obviously if the number of the lampposts of the isle is higher, the break-even will be reached earlier.

### 7.6. Management of Lamp Post Faults

In case of lamp post faults, to accelerate restore operations, the system has to inform the users as quickly as possible. To do it, the system has both a ZigBee communication control and a voltage battery one. The first checks both the breaking of the ZigBee element and the electronic card. In both cases the program shows, on its graphical interface, the problem. The system also checks the supply coming from the battery. If it goes down, a 4.5 V backup battery, integrated in the box, provides the necessary energy to work. The electronic card is moreover designed to check the backup battery voltage so, if this is too low, a battery fault signal will be sent to the coordinator program.

## 8. Comparative Studios

To shows the advantages of our SC application, three other analysis have been conducted. The first shows a comparison between our isle and another realized with classical technology with lamp posts without presence sensors and supplied by mains. Considering the month of Table 6, the consumption of the classical isle composed of five lamp posts would be equal to 145.75 kWh with an energy cost of 29.15 € and a carbon production equal to 77.25 kg [41]. Obviously, with our isle the cost and the production of carbon dioxide is equal to zero.

The second shows an extension of the proposed system to other two isles of lamp posts realized in the meantime. Both these isles are composed by five lamp posts and are always placed in rural areas. Again, for both, WiMAX is the only technology usable for the Internet connection. The months considered are only November and December 2014 in which the three isles were contemporaneously active. If the isles had been realized with conventional technology, the overall power consumption was about 220 kWh with a cost of 44 € and a production of 116.6 kg of CO_2_. Always in this case using our system the cost and the production of carbon dioxide is equal to zero.

Considering an annual use of the three isles equals about 67,725 kWh, the total estimated savings is equal to about 13,545 € and a production of about 35,894 kg of CO_2_. It easy imaginable that a bigger number of isles allows one not only to increase the savings, but also to reduce the overall maintenance costs thus increasing the benefits.

Another comparison has been realized changing the scheme of the isle connecting in series eight lamp posts located 20 m away from each other. In this configuration, the presence sensor cards are placed at the start and at the end of the line. The overall functioning of the system is similar to the previous configuration, but, in this case, a transmission data problem surely happens. In fact, the ZigBee transmission range of XBee module is at maximum 100 m in LOS, so this obliges us to transform the mesh ZigBee architecture into a serial architecture where data sent by the n-lamp post, must be acquired by the n−1-lamp post, and so on up to the Coordinator. To face the problem it is necessary to modify the Monitoring Station Software creating a recursive program able to pack the data coming from the n-lamp post and send them together with data of the n−1-lamp post towards the n−2-lamp post and so on up to the Coordinator. On the other side, the requests from the Coordinator toward the secondary lamp posts have to be similarly packed and serially delivered to the lamp posts. Making these changes, the system correctly works. From an economical point of view, the general considerations made for a mesh configuration are equally valid.

## 9. Conclusions

The application of new technologies to a system, historically not the subject of much innovation, can transform it into an extremely efficient system allowing energy and money savings if compared with classical systems, as the Smart City paradigm teaches.

The example shown in this article is a clear demonstration of this thesis. An isle of lamp posts used to light a crossroads and placed far from the city where the Internet signal does not arrive, has been re-designed combining all new technologies available on the market: LEDs for the lamps, PV panels for the power supply and an intelligent management.

This last foresees an architecture that uses local sensors for intelligent lighting of the lamp, the storage of the functioning data, and their sharing by a local communication wireless mesh realized by ZigBee devices that send information to the coordinator lamp equipped with a RaspBerry-Pi card. The RaspBerry-Pi has been chosen for its low costs and for the possibility to drive also a WiMAX modem/router which allows to make the data system visible by a web site accessible by Internet also for areas very far from the city and not reached neither by the ADSL line nor by 3G signals.

Some analysis and comparisons testify the validity of the adopted choices. For its reliability, simplicity and low cost, the proposed system can also be used to update existing conventional lamp posts making it a serious candidate to efficiently manage a set of sensors applicable in different fields including monitoring of energy consumption, other Smart City application and smart grids which needs to diffuse sensors and actuators to realize an efficient management of the system under control.

## Figures and Tables

**Table 1.** ZigBee *vs.* some other wireless network.

	**ZIGBEE**	**WI-FI**	**BLUETOOTH**
IEEE standard	802.15.04	802.11b/g	802.15.01
Main application	Control	Broadband	Mobile devices
Number of network devices	Up to 65,000	32	7
Bit rate	20–250 kb/s	11/54 Mb/s	720 kb/s
Range	100 m	100 m	10 m
Battery life	100–1000 days	1–5 days	1–7 days

**Table 2.** Raspberry-Pi *vs.* Arduino and Beaglebone.

	**RaspBerry**	**Arduino**	**Beaglebone**
Base Price in US $	25	35	50
Power Draw	∼150 mA @ 5V	∼50 mA @ 5V	∼250 mA @ 5V
Operating System	Linux	Custom	Linux
Suited for	Software	Hardware	Software
Number of I/O pins	8 Digital	14 Digital (6 PWM), 6 analog	65 Digital
Peripherals	2 USB Hosts, 1 Micro-USB Power, 1 10/100 Mbps Ethernet	None	1 USB Host, 1 Mini-USB Client, 1 10/100 Mbps Ethernet
Internet	Yes	*Via* Shield	Yes

**Table 3.** WiMAX *vs.* other communication technologies.

	**WiMAX**	**ADSL**	**WiFi**	**3G**	**Satellite Communication**
Wireless?	Yes	No	Yes	Yes	Yes
Distance Covered	Up to 70 km from the CPE	Everywhere with cable	Up to 100 m	Up to 5 km from the BS	Everywhere the signal satellite is on
Subscription Costs	About 20 €	About 20 €	About 20 €	About 20 €	About 100 €
Availability in rural area	Medium/High	No	No	Medium	Yes

**Table 4.** ZigBee reliability tests.

**XBEE STANDARD—Patch Antenna**		
	**Sunny**	**Rainy**
	**up to 45 m**	**up to 45 m**
No obstacles	100%	100%
Vegetation	100%	100%

**Table 5.** WiMAX uplink modulation in LOS.

**UL Modulation**	**Packet Loss**	**CINR (dB)**	**AvgDistance from BS (km)**
16-QAM3/4	0%	23	4.5
16-QAM1/2	0%	15	7.3
QPSK3/4	0%	13	10.1
QPSK1/2	0%	9	12.9

**Table 6.** Current consumption and operating time of new and old lampposts.

**Lamp ID**	**February**		**kWh**	**Cost h × kWh (0.200 €)**	**Plant Costs**					
	
	**Time (h)**	**I (A)**			**Battery Type and Cost**	**Solar Panel Type and Cost**	**Battery Recharger**	**Lamp €**	**Power Supplier**	**Electronic Devices €**
New Lamp (L1)	21.36	1.48	0.367	0	5 A–16 €	19 W–50 €	10 €	18	0	40

New Lamp without light sensor (L2)	280	1.49	5.006	0	20 A–25 €	80 W–220 €	10 €	18	0	0

New Lamp without presence sensor and supplied by mains (L3)	280	0.09	5.796	1.159	None	None	0	18	30	0
